# Effect of School Closure from Pandemic (H1N1) 2009, Chicago, Illinois, USA

**DOI:** 10.3201/eid1704.100906

**Published:** 2011-04

**Authors:** Vanessa G. Jarquin, David B. Callahan, Nicole J. Cohen, Victor Balaban, Rose Wang, Ricardo Beato, Paran Pordell, Otilio Oyervides, Wan-Ting Huang, Harvey Lipman, Daniel Fishbein, Mehran S. Massoudi

**Affiliations:** Author affiliation: Centers for Disease Control and Prevention, Atlanta, Georgia, USA

**Keywords:** Influenza, viruses, school closure, mitigation, Chicago, letter

**To the Editor:** On April 28, 2009, the Chicago Department of Public Health received notification of 1 student at an elementary school with a probable pandemic (H1N1) 2009 virus infection; the infection was subsequently laboratory confirmed. This case was one of the first pandemic (H1N1) 2009 cases in Chicago. To prevent transmission of influenza and with guidance from the Chicago Department of Public Health, the school closed on April 29; it reopened on May 6 after the Centers for Disease Control and Prevention (CDC) revised its recommendations (*1*). We conducted an investigation to evaluate psychosocial and economic effects of the school closure on the students' families and to assess whether students complied with mitigation recommendations. In the early pandemic, Chicago’s number of pandemic (H1N1) 2009 cases was one of the highest in the United States (*2*).

Households were surveyed if >1 child in the household was enrolled in the school and contact was made with an adult (parent/guardian). We made a minimum of 3 attempts to contact eligible households by telephone in English or Spanish. Households without working telephone numbers were visited, but only 1 visit yielded a completed interview. The school had an enrollment of 744 students (609 households, of which 439 were reachable by telephone) during April–May 2009. The final sample comprised 170 households (39% of reachable households). Fifty-four (31%) respondents were employed full-time and 37 (22%) part-time; 78 (46%) were unemployed, homemakers, students, or retired. Households had a median of 2 adults and 2 children in grades prekindergarten through eight.

In contrast with findings of Johnson et al. (*3*) in an investigation of an influenza B virus outbreak, where 89% of students visited >1 public location during the school closure, results from our investigation (Table) indicate that most students complied with recommended social distancing measures. Johnson et al. highlighted the potential for transmission in public areas during a school closure. However, with only approximately one third of households in this investigation reporting their children went to public areas during the school closure, the same level of concern of public transmission was not found.

**Table:** Household responses (n = 172) related to school closure as a result of pandemic (H1N1) 2009, Chicago, Illinois, USA, April 29–May 5, 2009

Response	No. (%)
Highest education level of parent or guardian	
None	2 (1)
Elementary school	48 (28)
Junior high school	9 (5)
High school	59 (34)
Some college	29 (17)
Advanced degree	21 (12)
No response	4 (2)
Employment status of parent or guardian	
Full time	54 (31)
Part time	37 (22)
Student	6 (4)
Retired	2 (1)
Unemployed	17 (10)
Stay-at-home	53 (31)
Other/no response	3 (2)
Receipt of closure information by parent or guardian*	142 (84)
School	89 (63)
Radio or television news	81 (57)
Other parents/students	17 (12)
Student	5 (4)
Press conference	4 (3)
Internet	3 (2)
Found closure difficult for self or family*	105 (61)
Fear about H1N1	74 (70)
Uncertainty about duration of closure	70 (66)
Fear about family’s health	66 (62)
Schedule changes	33 (31)
Student missing school meals	26 (25)
Child care arrangements	21 (20)
Loss of income because of lost work time	17 (16)
High cost of child care arrangements	13 (12)
Transportation difficulties	12 (11)
Student missing education	3 (3)
Behavioral concerns related to disability	1 (1)
Student activities during closure*	
Did homework	125 (73)
Went to a public place	63 (37)
Went to home of another family member	43 (25)
Got together with <6 friends	29 (17)
Went to afterschool extracurricular activity	20 (12)
Got together with >6 friends	13 (8)
Slept at a friend’s house	5 (3)
Went to afterschool program	5 (3)
Alternate child care arrangements made†	13 (8)
*Response categories were not mutually exclusive. †Mean cost of alternate childcare $45.	

The results from this investigation indicate the economic effect of the school closure was minimal for survey respondents. These results were similar to those found by Johnson et al. (*3*), which had only 18% from 220 households (with 315 employed adults) report missing work to stay home because of school closure. However, the number of families losing work time in our investigation was much lower than the 53% of families in central Virginia reported by Nettleman et al. (*4*) using a survey of school absenteeism and employment status for adults who stayed home to care for an ill child. This might have been because 31% of respondents surveyed in this investigation were homemakers, and an additional 10% were unemployed or retired. Therefore, many parents and legal guardians from this investigation did not need to noticeably change their daily routine to care for their children during the closure. Moreover, compliance has been shown to vary by income and employment status (*5*).

CDC guidance issued on April 27, 2009, recommended closing any school that had a laboratory-confirmed case of pandemic (H1N1) 2009 (*1*). As new information became available, CDC updated its recommendations, reflecting consideration of the overall benefits and harms, including students being left home alone, parents missing work to care for their children, students missing meals, and students’ education being interrupted (*1*). The findings from investigating the effect of this school closure support other CDC recommendations and are relevant for future pandemics.

Our study was limited by the low household participation rate, which might have biased the current findings. However, student characteristics, including race/ethnicity, grade level, and enrollment in free/reduced lunch and special education services received, were consistent with demographics of the school (*6*).

This relatively brief school closure had limited effect on the families in our study, but a school closure in a different community, at a different time, or perhaps of longer duration than 1 week might have a greater effect and prove to be more difficult for parents. The public health benefits of future school closure might increase if strategies were implemented to increase students’ compliance with recommendations to avoid public places or group gatherings to decrease exposure to pandemic (H1N1) 2009 and seasonal influenza. In addition, parent education on infection control strategies is necessary to increase compliance. However, strategies should limit the disruption to day-to-day activities of families and learning in the schools. Interruptions in school lunch programs might be offset by providing meals in noncongregate settings outside of school or involving community organizations. Further research is needed to understand the economic effect and timing of school closures in other populations or communities, and to understand the efficacy of school closure on reducing transmission of other communicable diseases.
